# The Tibial Plateau fracture—Current incidence and treatment in Germany

**DOI:** 10.1371/journal.pone.0323443

**Published:** 2025-05-09

**Authors:** Julia Elisabeth Lenz, Lorenz Huber, Josina Straub, Wolf Bäumler, Volker Alt, Johannes Weber

**Affiliations:** 1 Department of Trauma Surgery, University Medical Center Regensburg, Franz‑Josef‑Strauss Allee 11, Regensburg, Germany; 2 Department of Radiology, University Hospital Regensburg, Franz‑Josef‑Strauss Allee 11, Regensburg, Germany; Carol Davila University of Medicine and Pharmacy: Universitatea de Medicina si Farmacie Carol Davila din Bucuresti, ROMANIA

## Abstract

**Purpose:**

This study aims to analyze the epidemiology, incidence, and treatment of tibial plateau fractures in Germany from 2019 to 2022. The focus is on understanding demographic trends, clinical presentations, and surgical management strategies for this severe injury.

**Methods:**

A retrospective cohort study was conducted using data from the German Institute for the Hospital Remuneration System (InEK) from 2019 to 2022. Cases were identified using the International Classification of Diseases 10th Revision (ICD-10) codes related to tibial plateau fractures, and documented surgical procedures were categorized using OPS codes. The Patient Clinical Complexity Level (PCCL) was used to assess the severity of cases.

**Results:**

A total of 79,158 cases of tibial plateau fractures were recorded during the study period, with an incidence of 22.4–25.3 per 100,000 inhabitants. Women were more frequently affected, accounting for 61.5% ± 1.1% of cases annually. The average hospital stay was 9.2 ± 0.1 days, and 76.5% ± 0.5% of patients were categorized at PCCL 0. Most fractures were multifragmentary 68.8% ± 1.3%, and the predominant documented treatment method was open reduction and internal fixation with plate osteosynthesis 63.5% ± 2.8%. 23.6% ± 2.2% of cases required bone grafting, with a preference for allografts.

**Conclusions:**

Tibial plateau fractures represent a significant and stable burden in Germany, with an incidence ranging from 22.4 to 25.3 per 100,000 inhabitants. Women accounted for 61.5% ± 1.1% of cases, highlighting a gender-related predisposition. The mean hospital stay remained stable at 9.2 ± 0.1 days. The study underscores the need for tailored treatment strategies and highlights the importance of preventive measures, particularly for the elderly population, in order to reduce the incidence of these fractures. Future research should focus on improving surgical techniques and postoperative care to enhance patient outcomes and potentially reduce hospital stay durations.

## Introduction

Tibial plateau fractures represent severe injuries involving the knee joint. Although these fractures constitute a small percentage of orthopedic injuries, their complexity make them a critical focus in orthopedic trauma care [1].

A tibial plateau fracture occurs when there is a break in the proximal part of the tibia, impacting knee stability and function. These injuries are frequently associated with ligament and meniscus damage, leading to long-term impairment if not properly managed [2].

Risk factors include high-energy trauma, such as motor vehicle accidents and falls from heights, as well as low-energy fractures in osteoporotic elderly patients [3].

Severity is determined by joint affection, fragment displacement, and associated soft tissue injuries [4,5]. The Schatzker classification system is commonly utilized to categorize tibial plateau fractures from simple splits to complex comminuted fractures [6]. Further classification tools include those published by the “Arbeitsgemeinschaft für Osteosynthesefragen” (AO), Duparc et al, Hohl et al and Luo et al [7–11].

Management typically involves initial stabilization, followed by assessment of the fracture pattern and soft-tissue injuries [3]. Treatment ranges from non-operative methods like bracing and physical therapy to surgical interventions aimed at restoring joint alignment and stability [4]. In cases of significant joint depression or instability, open reduction and internal fixation (ORIF) is often required [12].

Arthroscopic techniques have emerged as valuable adjuncts in tibial plateau fracture treatment, allowing for minimally invasive reduction, better intra-articular visualization, and improved soft tissue preservation [13–17]. Recent advancements focuse on three-dimensionally modeled minimally invasive rim plate osteosynthesis techniques to optimize the fixation of posterolateral tibial plateau fractures [18] and staged management approaches to minimize soft tissue complications in high-energy fractures [19].

For bone defects, allogenous, autogenous, or synthetic bone grafting may be necessary. Total knee arthroplasty is an option for severely damaged joints, particularly in geriatric patients [20–23].

Given the potential for significant morbidity, tibial plateau fractures require a comprehensive, multidisciplinary, and well-timed approach to treatment [24]. The primary objectives of treatment include stabilizing the joint, restoring function, and preventing further complications such as post-traumatic arthritis.

This study aims to provide detailed information about the epidemiology and incidence of tibial plateau fractures in Germany from 2019 to 2022 and an overview of current treatment practices.

## Materials and methods

This retrospective cohort study examines all cases of tibial plateau fractures from 2019 to 2022 as well as all documented surgical treatments of tibial plateau fractures conducted at German medical institutions from 2019 to 2022, as reported by the German Institute for the Hospital Remuneration System (InEK – Institut für das Entgeltsystem im Krankenhaus).

The study included all tibial plateau fractures treated in German hospitals with insurance coverage, encompassing all German residents as well as internationally insured patients.

The study utilized patient data associated with the International Classification of Diseases 10th Revision (ICD-10) codes for “tibial plateau fracture” (S82.11, S82.18) to identify hospitalized patients with this condition over the four-year period. This enabled a comprehensive epidemiological analysis focusing on age and sex distribution. The type of surgical procedure performed was reported using operation and procedure codes (OPS-Codes). A minimum of five patients per group was required for inclusion in the InEK registry.

The extracted data included patient demographics (age, sex), fracture characteristics (multifragmentary or simple), associated injuries (ligament, meniscus, soft tissue, and neurovascular injuries). Additionally, we extracted information on the prevalence of obesity and comorbidities, hospital length of stay, and the Patient Clinical Complexity Level (PCCL). For operatively treated patients, the type of treatment received (open reduction and internal fixation, screw osteosynthesis, external fixation, or arthroscopy) was extracted.

All diagnoses of tibial plateau fractures from 2019 to 2022 were included in the study analysis concerning the demographic data, and all patients with a documented operative treatment were included in the analysis of operative treatments. The InEK also reported the Patient Clinical Complexity Level (PCCL), which is determined through a complex procedure based on secondary diagnosis values, indicating the severity of complications or comorbidities on a scale from 0 (low complexity) to 6 (high complexity) [25,26]. The classification system for soft tissue injuries used was the one proposed by Tscherne et al [27].

Categorical data are presented as frequency counts (percentages) and mean values with standard deviations. Data analysis was performed using the statistical software SPSS Version 26.0 (IBM, SPSS Inc., Armonk, NY, USA).

## Results

The number of tibial plateau fracture cases included in this study were 21,051 in 2019, 19,326 in 2020, 18,595 in 2021, and 20,186 in 2022, resulting in 79,158 cases in total. This results in an incidence of 22.4–25.3/ 100.000 inhabitants in Germany.

Women were more frequently affected by tibial plateau fractures than men, with female patients accounting for 61.5% ± 1.1%.

The average length of stay for patients was 9.2 ± 0.1 days.

**Fig 1:**
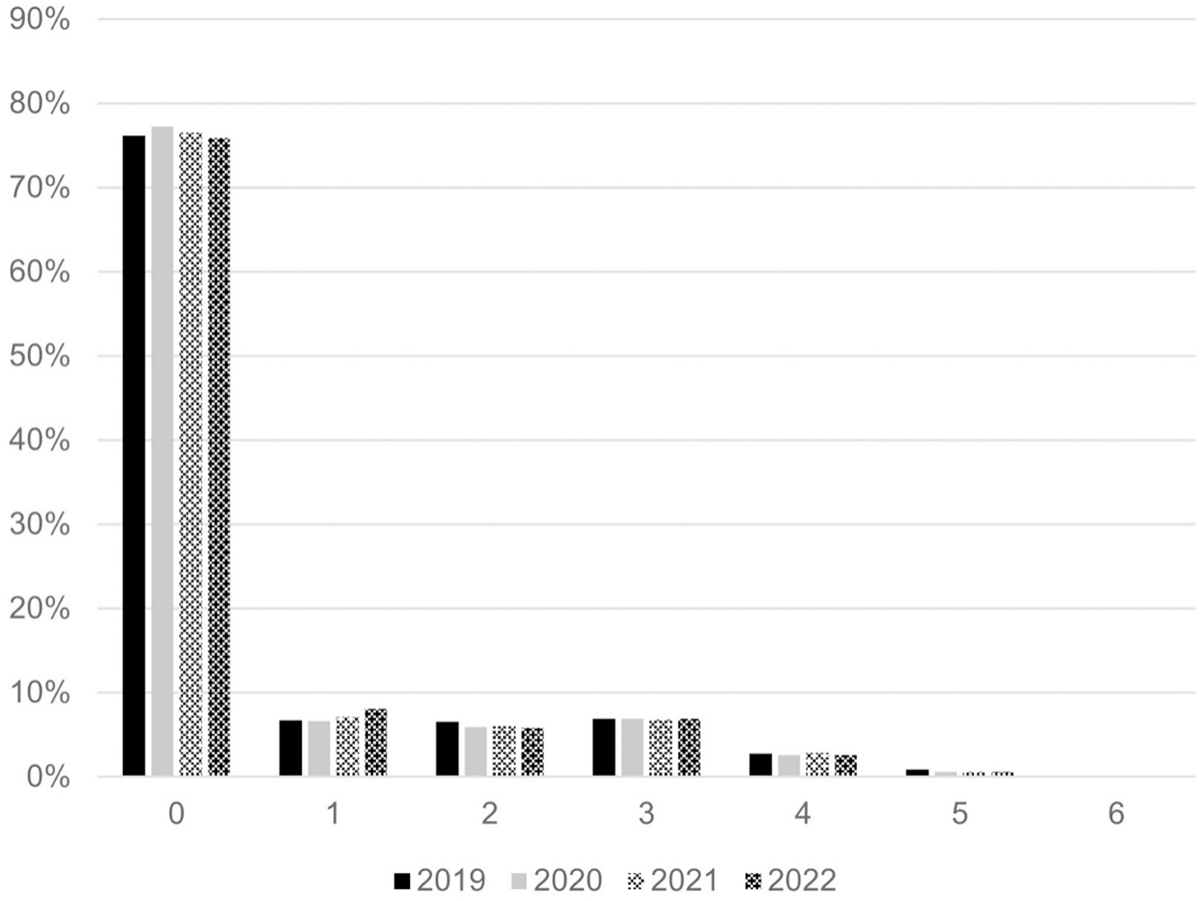
Patient Clinical Complexity Level (PCCL). Percentages are given as the respective patient proportion in relation to the total patients included in each year.

A mean of 76.5% ± 0.5% of patients were categorized at Patient Clinical Complexity Level PCCL 0 (see Fig 1).

**Fig 2:**
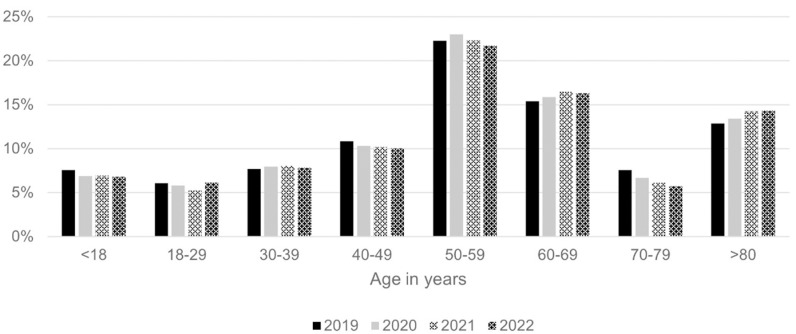
Patients‘Age Distribution. Percentages are given as the respective patient proportion in relation to the total patients included in each year.

The incidence of tibial plateau fractures increased with age (see Fig 2).

**Fig 3:**
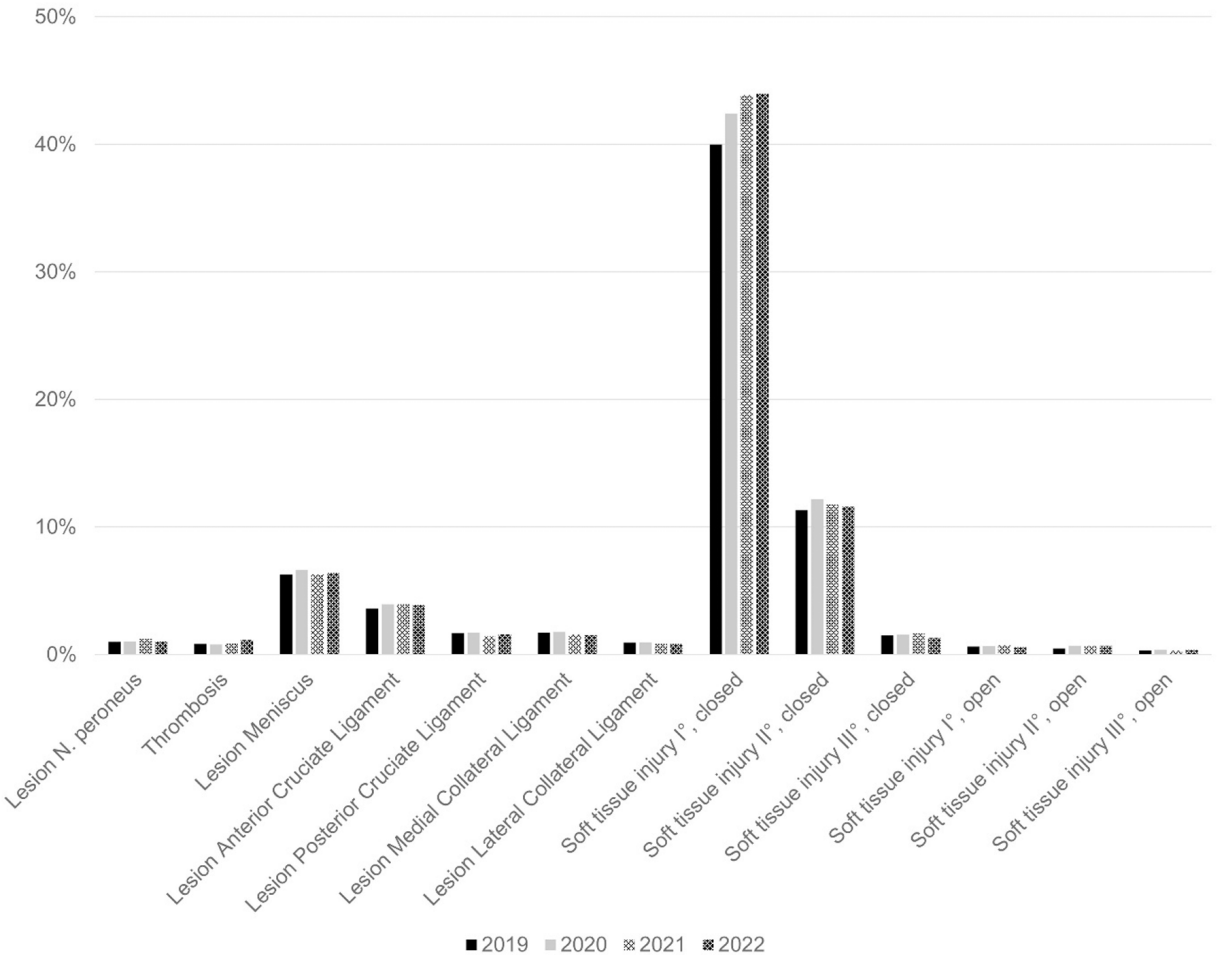
Concomitant injuries. Percentages are given as the respective patient proportion in relation to the total patients included in each year.

Most frequent accompanying injuries can be divided into ligament and meniscus injuries, soft tissue injuries and neurovascular lesions (see Fig 3). The most frequent injuries observed were first-degree closed soft tissue injuries, affecting 42.6% ± 1.6% of patients. These were followed by second-degree closed soft tissue injuries, occurring in 11.7% ± 0.3% of cases. 6.4% ± 0.1% of patients experienced a concomitant meniscus lesion, while 3.8% ± 0.1% presented with an anterior cruciate ligament rupture. Ruptures of the posterior cruciate ligament were noted in 1.6% ± 0.1% of patients. Collateral ligament lesions, thrombosis, and nerve damage were infrequent.

2.8% ± 0.8% of all patients had Grade I obesity, 1.8% ± 0.1% had Grade II obesity and 1.4% ± 0.1% had Grade III obesity.

OPS-Codes and therefore operative treatment were documented in 72.0% of cases in 2019, 79.1% in 2020, 80.0% in 2021 and 85,5% 2022.

**Fig 4:**
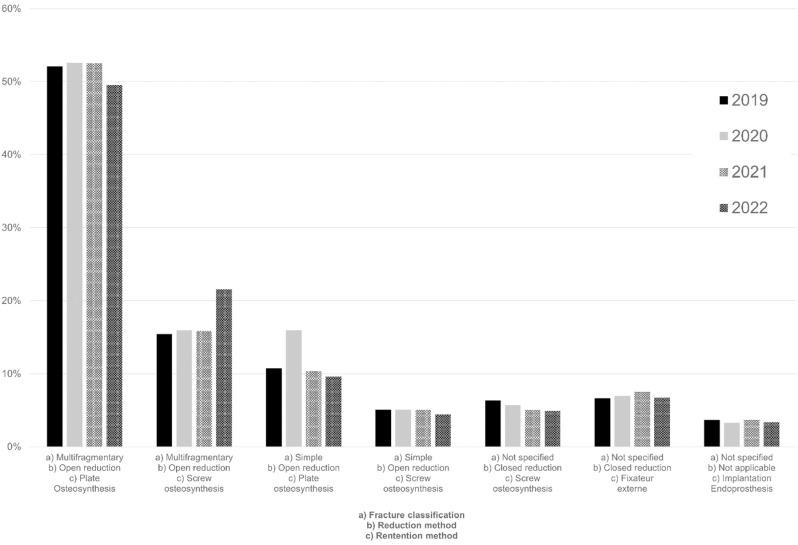
Fracture treatment. Percentages are given as the respective patient proportion in relation to the total patients with recorded fracture treatment per year.

Documented procedures performed were most often plate and screw osteosynthesis, according to AO standards (see Fig 4). Fractures have been classified as multifragmentary in 68.8% ± 1.3% of cases, while 16.57% ± 2.5% have been identified as simple fractures. In 15.9% ± 3.5% of cases, a fracture classification was not comprehensible. Closed reduction was carried out in 12.4% ± 0.8% of cases. A fixateur externe was applied in 42.9% of these cases following closed reduction, in intention of damage control surgery and/or soft-tissue conditioning, while 57.1% of patients underwent percutaneous screw osteosynthesis.

For both multifragmentary and simple fractures, open reduction with plate osteosynthesis was the preferred method of stabilization, accounting for 63.5% ± 2.8%of cases. 27.6% ± 2.6% of patients received a screw osteosynthesis.

**Fig 5:**
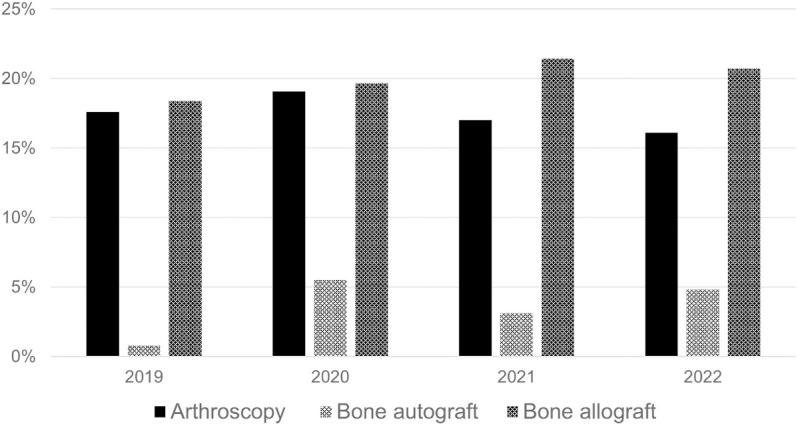
Adjunctive Procedures. Percentages are given as the respective patient proportion in relation to the total patients included in each year.

Arthroscopy was conducted in 17.5% ± 1.1% of operations (see Fig 5). Bone grafting was conducted in 23.6% ± 2.2% of cases- with the vast majority of 85.6% ± 6.8%utilizing allografts. Autografting was seldomly performed.

**Fig 6:**
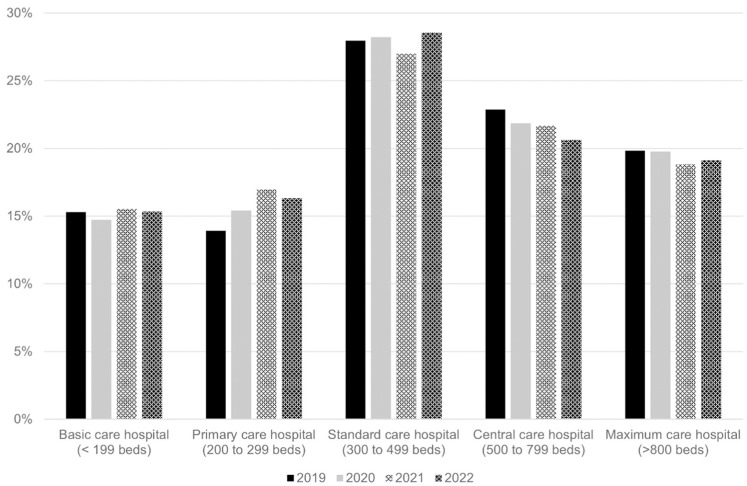
Type of hospital. Percentages are given as the respective patient proportion in relation to the total patients included in each year.

The distribution of hospital types is uniform, with a peak observed at standard care hospitals, where 27.9% ± 0.6% of patients were treated (see Fig 6).

## Discussion

This epidemiological study on tibial plateau fractures in Germany from 2019 to 2022 provides an extensive analysis of a significant orthopedic injury: with a total of 79,158 cases analyzed, the study highlights the considerable burden these fractures impose on the healthcare system and identifies important trends in their incidence, demographics, clinical presentation, and management.

The incidence of tibial plateau fractures in Germany, ranging from 22.4 to 25.3 per 100,000 inhabitants, underscores the frequency with which these injuries occur [28,29]. The relatively stable incidence rate over the last four years suggests that the underlying risk factors, such as falls and motor vehicle accidents with axial loading combined with valgus or varus stress, remain consistent within the population [30–32].

However, data also reveals a significant sex disparity, with women consistently representing a higher percentage of cases from 59.5% in 2019 rising to 62.5% by 2022. This sex difference is likely due to several factors, including the higher prevalence of osteoporosis in women, particularly postmenopausal women, which increases their susceptibility to fractures from low-energy impacts [33,34]. The slightly increasing proportion of female patients over the study period may also reflect broader demographic trends, such as an aging female population with longer life expectancy.

The age distribution of patients indicates that the incidence of tibial plateau fractures increases with age, which aligns with the understanding that older individuals are more vulnerable to such injuries due to decreased bone density and increased fall risk [28,33,34]. Moreover, two distinct peaks can be identified: one corresponding to young male patients and the other to older female patients, aligning with the high- and low-impact model. This trend has significant implications for public health and preventive strategies, emphasizing the need for targeted interventions to reduce the risk of falls and fractures among the elderly.

The average length of hospital stay for patients with tibial plateau fractures remained consistent at about 9.2 days. These numbers are consistent with literature and suggest that, despite advancements in surgical techniques and postoperative care, the overall recovery time for these injuries has not significantly changed [35–37]. Fast-track concepts and earlier mobilization should therefore be considered, as these approaches have proven successful in comparison to total joint replacements with shorter hospital stays [38].

The Patient Clinical Complexity Level (PCCL) data, showing that approximately 76% of patients were categorized at PCCL 0, indicates that the majority of patients did not present with severe comorbidities. This finding may help explain the stable length of hospital stay observed, as patients with lower clinical complexity tend to have shorter and more predictable recovery periods.

The relatively low frequency of obesity and comorbidities in our cohort compared to the general population may be attributed to selection bias, as healthier individuals with fewer chronic conditions are more likely to experience high-energy trauma leading to tibial plateau fractures. Additionally, underreporting in administrative databases is a well-documented limitation that can lead to an underestimation of comorbidity prevalence [39,40].

The patterns of associated injuries reveal that closed soft tissue injuries were the most common concomitant injuries. These were followed by second-degree closed soft tissue injuries. The occurrence of meniscal and ligamentous injuries, such as meniscus lesions in approximately 6% of patients and anterior cruciate ligament ruptures in around 4%, reflects the complex nature of the trauma that often accompanies tibial plateau fractures. The relatively low incidence of posterior cruciate ligament ruptures and collateral ligament injuries is consistent with the typical biomechanical forces involved in these fractures, where axial loading combined with valgus or varus stress primarily affects the tibial plateau and adjacent soft tissues [41,42].

Surgical management of tibial plateau fractures was predominantly characterized by open reduction and internal fixation, with plate osteosynthesis being the preferred method of stabilization in both multifragmentary and simple fractures. This approach of osteosynthesis procedures following open reduction, aligns with AO principles for periarticular fractures or fractures involving the epiphyseal region, emphasizing the importance of stable fixation to facilitate early mobilization and minimize the risk of complications [43].

Percutaneous screw osteosynthesis represents a less invasive option that is primarily utilized for split fractures, accounting for its rare application. The selective use in these specific fracture patterns, which typically lack extensively depressed comminuted zones, contributes to the promising outcomes observed. A systematic review revealed that over 85% of patients treated with this method achieved excellent or good results according to clinical and radiological Rasmussen scores. The overall complication rate remains low at 6.6%, with loss of reduction being the most common issue at 2.4%. This is particularly relevant in highly comminuted fractures with depressed fracture zones, where the complexity of the injury may increase the risk of complications [15].

Approximately 3.5% of patients were treated with knee endoprosthesis implantation per year. In patients with fractures of the tibial plateau, the risk for indication of implantation of a total knee arthroplasty is increased due to posttraumatic arthritis [20]. Primary total knee arthroplasty (TKA) for tibial plateau fractures demonstrates good outcomes, particularly in geriatric patients by enabling early mobilization and potentially reducing the need for reoperation, although it does not consistently surpass the efficacy of open reduction and internal fixation (ORIF) [20]. The conversion to secondary TKA following tibial plateau fracture treatment occurs in approximately 5.1% of cases, with the highest incidence within the first five years post-fracture [21]. Risk factors for conversion include female sex, advanced age, and treatment in low-volume surgical centers. Notably, secondary TKA is associated with significantly higher complication and revision rates compared to primary TKA, which itself has higher rates than those observed in elective primary TKA [22,44].

Arthroscopy was performed in 17.5% ± 1.1% of operations. Arthroscopy-assisted reduction and internal fixation (ARIF) serves as a valuable adjunctive technique for certain fracture patterns, offering advantages such as faster postoperative recovery, improved clinical function, and the ability to address additional intra-articular lesions. While radiological outcomes and complication rates are comparable between ARIF and traditional open reduction internal fixation (ORIF), ARIF is particularly beneficial for its potential to enhance recovery in appropriately selected cases [13,14,45].

Bone grafting was conducted in 23.6% ± 2.2% of cases, with a strong preference for allografts (85.6% ± 6.8%) over autografts due to lower morbidity and fewer complications. Recent studies indicate that allograft and synthetic alternatives are non-inferior to autografts while reducing blood loss and shortening surgery times [46–48]. Integration rates are high and show no significant differences compared to autografts or the standard of care in treating fracture fragments [49,50].

To enhance patient outcomes and potentially reduce hospital stay durations following tibial plateau fractures, future research should focus on refining surgical techniques and postoperative care strategies. Integrating three-dimensional (3D) technologies into preoperative planning and surgical execution has been shown to improve the precision of fracture reductions, leading to better alignment and potentially shorter rehabilitation periods. Emphasizing early mobilization protocols post-surgery is also crucial, as they can enhance functional recovery and decrease the length of hospital stays [51,52].

The distribution of hospital types where patients were treated was uniform across all categories, with no significant trends favoring one type over another. This even distribution reflects the frequency of tibial plateau fractures as a standard injury, rather than a complex condition requiring treatment exclusively at specialized centers, highlighting the accessibility of appropriate care across all levels of healthcare institutions in Germany.

Our study has several limitations. One major drawback of all registry studies is that the analysis relies on the coding of diseases via International Classification of Diseases 10th Revision (ICD-10) and procedures (OPS). Errors in coding, such as misclassification as well as no classification at all, could not be identified. Therefore, the percentage of operatively treated patients cannot be determined with certainty. However, the data provided contains extensive information about all patients treated for tibial plateau fracture in German hospitals within the specified period. Another limitation is that treatment details could not be closely correlated with patient data, such as comorbidities or ASA scores, preventing risk and outcome analysis.

## Conclusion

Tibial plateau fractures represent a significant and stable burden in Germany, with an incidence ranging from 22.4 to 25.3 per 100,000 inhabitants. Women accounted for 61.5% ± 1.1% of cases, highlighting a gender-related predisposition. The mean hospital stay remained stable at 9.2 ± 0.1 days. The findings also highlight the importance of preventive measures, particularly in the elderly population, to reduce the incidence of these fractures. Future research should focus on refining surgical techniques and postoperative care protocols to further improve patient outcomes and potentially reduce the length of hospital stays.

## Abbreviations

AO: Arbeitsgemeinschaft für Osteosynthesefragen
ARIF: Arthroscopy-assisted reduction and internal fixation
ICD-10: International Classification of Diseases 10th Revision
InEK: German Institute for the Hospital Remuneration System
OPS: Operation and Procedure Codes
ORIF: Open reduction and internal fixation
PCCL: Patient Clinical Complexity Level.
